# The Impact of Heat Stress on Morpho-Physiological Response and Expression of Specific Genes in the Heat Stress-Responsive Transcriptional Regulatory Network in *Brassica oleracea*

**DOI:** 10.3390/plants10061064

**Published:** 2021-05-26

**Authors:** Mahdi Moradpour, Siti Nor Akmar Abdullah, Parameswari Namasivayam

**Affiliations:** 1 Laboratory of Agronomy and Sustainable Crop Protection, Institute of Plantation Studies, Universiti Putra Malaysia, Serdang 43400 UPM, Selangor, Malaysia; mahdimoradpour1@gmail.com; 2 Department of Agriculture Technology, Faculty of Agriculture, Universiti Putra Malaysia, Serdang 43400 UPM, Selangor, Malaysia; 3 Department of Cell and Molecular Biology, Faculty of Biotechnology and Biomolecular Science, University Putra Malaysia, Serdang 43400 UPM, Selangor, Malaysia; parameswari@upm.edu.my

**Keywords:** heat stress, physiological measurements, morphological characteristics, DPB3-1, cabbage breeding, gene expression, transcriptional regulatory network, heat stress response, thermotolerance

## Abstract

Knowledge of heat-tolerant/sensitive cultivars based on morpho-physiological indicators and an understanding of the action and interaction of different genes in the molecular network are critical for genetic improvement. To screen these indicators, the physiological performance of two different varieties of white and red cabbages (*B. oleracea* var. capitate *f. alba* and *f. rubra*, respectively) under heat stress (HS) and non-stress (NS) was evaluated. Cultivars that showed considerable cell membrane thermostability and less reduction in chlorophyll content with better head formation were categorized as the heat-tolerant cultivars (HTC), while those with reduction in stomatal conductance, higher reduction incurred in chlorophyll and damage to thylakoid membranes are categorized as the heat-sensitive cultivars (HSC). Expression profiling of key genes in the HS response network, including *BoHSP70* (HEAT SHOCK PROTEIN 70), *BoSCL13* (SCARECROW-LIKE 13) and *BoDPB3-1* (transcriptional regulator DNA POLYMERASE II SUBUNIT B3-1 (DPB3-1))/NUCLEAR FACTOR Y SUBUNIT C10 (NF-YC10), were evaluated in all cultivars under HS compared to NS plants, which showed their potential as molecular indicators to differentiate HTC from HSC. Based on the results, the morphophysiological and molecular indicators are applicable to cabbage cultivars for differentiating HTC from HSC, and potential target genes for genome editing were identified for enhancing food security in the warmer regions of the world.

## 1. Introduction

Many commonly cultivated *Brassica* vegetables such as cabbage, broccoli, cauliflower, kale and brussels sprouts belong to the *Brassica oleracea* species [1]. They are low-calorie and serve as a rich source of glucosinolates and carotenoids, as well as other vitamins, minerals and anthocyanins that are beneficial to human health [2,3]. However, *Brassica* vegetables are cool season crops, and most varieties are highly vulnerable to high temperature which stimulates various and often incompatible changes in plant growth, development and physiological processes which together adversely affect crop yield [4].

However, plant response to high temperature varies with the degree of temperature increment, exposure time and the type of plant. At extremely high temperatures, there will be a rapid occurrence of damage in the cells that disrupts cellular organization [5]. Heat stress (HS) has an impact on different plant processes including germination, growth and developmental and reproductive mechanisms [6]. The stability of different RNA species, proteins, membranes and cytoskeleton structures will also be adversely affected. In addition, the efficiency of enzymatic reactions taking part in major physiological processes will be altered, creating metabolic imbalance [7,8,9]. In *Brassica* vegetables, the negative effects of high temperature include the production of narrow leaves, leaf growth reduction, delays in heading initiation and undesired petiole-to-blade ratio enhancement (e.g., in Chinese cabbage), together contributing to lower yields and poor marketability due to the reduction in quality [10]. Cabbage head formation, which is significantly influenced by the interplay between genotype and environment [11], serves as the main phenotypic index for heat tolerance in cabbage at high temperatures [2].

The retention of protein conformation and the prevention of non-native protein aggregation are especially critical for the survival of cells under stress [12]. Reactive oxygen species (ROS)-scavenging enzymes, non-enzymatic antioxidants and heat-shock proteins (HSPs) play crucial roles in protecting cells against stress, and they are regulated through a complex transcriptional regulatory network [2,13]. HSPs can be divided into five different families: HSP100, HSP90, HSP70, HSP60 and HSP20. Their expression is confined to certain plant developmental stages such as seed germination, embryogenesis and maturation. They stabilize proteins and membranes that help in protein refolding, providing protection against stress by re-establishing the normal conformation of proteins [12]. The high conservation of HSP70 and HSP60 proteins is also essential for their critical role in HS response [4]. They are induced by HS and are recognized target genes of HS-responsive transcription factors [14].

An extensive transcriptional network comprising many heat stress-responsive transcription factors and the different transcriptional regulators regulates the expression of HS-inducible genes [14]. TFs dehydration-responsive element binding protein 2A (DREB2A) triggers the expression of heat shock transcription factor A3 (HSFA3) as a direct target gene with a coactivator complex consisting of NF-YA2 (nuclear factor Y, subunit A2), NF-YB3 and DPB3-1/NF-YC10 (DNA polymerase II subunit B3-1 (DPB3-1))/nuclear factor y subunit c10 (NF-YC10) [15]. Prior investigations showed that DPB3-1 serves as a coactivator of DREB2A that acts only under abiotic stress. It was shown that DPB3 could be utilized to increase HS tolerance in transgenic plants without negative effects on vegetative and reproductive growth [16,17]. C3H4 RING domain-containing proteins, DRIP1 (dreb2a-interacting protein 1) and the DRIP2 were identified as DREB2A interactors that functions as E3 ubiquitin ligases mediator for DREB2A ubiquitination. Under non-stress condition (NS), double mutation of DRIP1/DRIP2 resulted in the increase in the wild-type DREB2A. DRIPs may perform as unique negative regulators involved in regulating the expression of drought-responsive genes by targeting DREB2A to 26S proteasome proteolysis [18,19].

The plant-specific GRAS transcription factor family are important plant-specific proteins, named based on the first three members: GIBBERELLICACID INSENSITIVE (GAI), REPRESSOR of GAI (RGA) and SCARECROW (SCR), which regulate diverse processes involved in plant growth, development and stress responses [20], although its function in the HS response is unclear [2]. Scarecrow-like protein 13 (SCL13), which belongs to the GRAS transcription factor family, serves as a positive regulator in the signal transduction by red light [21]. The suppression of SCL13 abated the sensitivity to red light in transgenic plants, indicating that SCL13 plays a role in the elongation of hypocotyls during de-etiolation. Gene-chip microarray gene expression studies suggested involvement of *BoHsp70* and *BoSCL13* in the tolerance to heat and the proper formation of cabbage heads under HS, and expression levels of *BoHSP70* and *BoSCL13* may perhaps be early selection markers for heat-tolerant cabbage cultivars in cabbage breeding. *BoSCL13* was thus proposed as a novel candidate gene useful for the identification of HS-tolerant cabbage with potential application in crop improvement programmes [2].

In this study, we evaluated the expression level of *BoDPB3-1* as a coactivator of *BoDREB2A* for the first time under HS treatments for the identification of heat-tolerant and heat-sensitive cabbage varieties. We also included *BoHSP70*, *BoSCL13* and *BoDRIP1* in our analysis as they are key genes in HS response pathways in cabbage with a potential impact on the morphological and physiological characteristics that influence thermostability during cabbage cultivation. Through our study, we were able to define the transcriptional network regulating HS response in cabbage to assist in future development of CRISPR-based crop modification and breeding for heat-tolerant cabbage cultivars.

## 2. Results: Comparison of Different *Brassica oleracea* Cultivars on Heat Stress Tolerance Using Morphological and Physiological Measurements 

### 2.1. The Morphological Differences between Heat-Sensitive Cabbage and Heat-Tolerant Cabbage Cultivars at High Temperature

**Figure 1:**
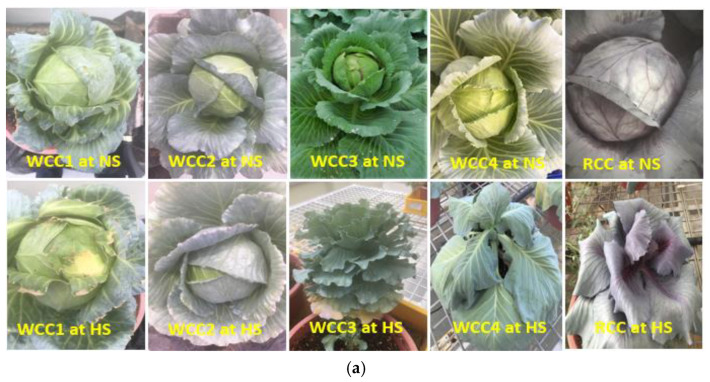

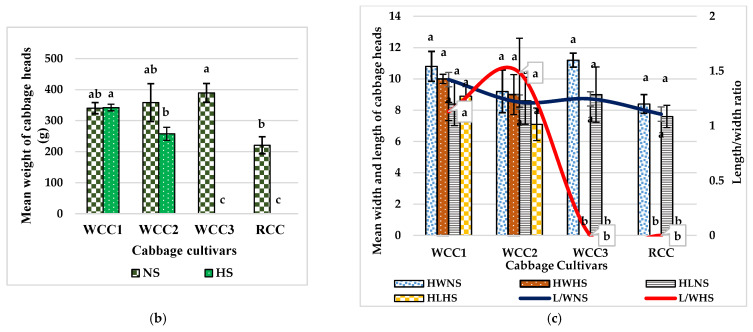
Effects of heat stress (HS) on morphological characteristics of different cabbage cultivars. Comparison of (**a**) HS phenotype of cabbage head formation, (**b**) mean head weight and (**c**) mean head width, length and their ratios. Bars denote the mean ± SE (*n* = 5). Mean values within each cultivar with different letters are significantly different based on Tukey’s HSD test at *p* < 0.05.

In this study, the effects of HS on the morphological characteristics of different white cabbage cultivars and one red cabbage cultivar, including head formation and mean head weight, width, length and their ratios, were measured and shown in Figure 1. Our results showed that all candidate cabbage cultivars for the morphological characteristics studies, including WCC1, WCC2, WCC3, WCC4 and RCC, were able to produce cabbage heads during NS, while WCC1 and WCC2 showed heat tolerance since they were able to produce cabbage heads during HS, whereas the WCC3, WCC4 and RCC represent the heat-sensitive phenotypes as they were not able to produce cabbage heads during HS (Figure 1a). WCC1 as a representative of heat-tolerant white cabbage cultivars, WCC4 as a representative of heat-sensitive white cabbage cultivars and RCC as a representative of heat-sensitive red cabbage cultivars were used as a positive or negative control for heat-shock stress in the gene expression studies. Furthermore, the mean weight of cabbage heads shows significant differences between white and red cabbage cultivars among cultivars under NS and HS (Figure 1b). However, among the same cultivars under the HS conditions (42 °C), only WCC1 and WCC2 formed cabbage heads, whereas WCC3 and RCC did not. In addition, the mean weight of cabbage heads among the candidate cultivars ranged from 342.1 g for WCC1 to 257.4 g for WCC2. It was noted that the mean weight of the cabbage heads of WCC1 NS and HS did not differ much. Moreover, the results obtained did not show any significant difference for the mean width and length of cabbage heads in both the heat-treated and untreated plants.

### 2.2. Effect of Heat Stress on Chlorophyll Content in Brassica oleracea Cultivars

**Figure 2:**
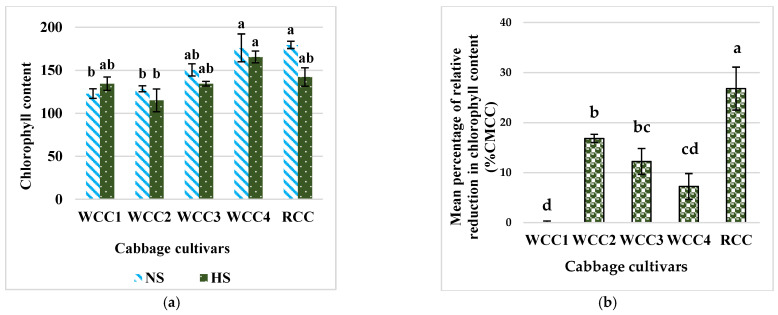
Mean phenotypic expression of chlorophyll content. (**a**) CM1000™ chlorophyll content index values and (**b**) mean percentage of relative reduction in chlorophyll content in five cabbage cultivars under HS (at 42 °C; for 5h) and NS (25 °C) conditions. Bars denote the mean of at least 5 measurements ± SE (*n* = 5). Mean values within each cultivar with different letters are significantly different based on Tukey’s HSD test at *p* < 0.05.

A large variation among cultivars was observed for the CM1000^TM^ chlorophyll index values (Figure 2a) under HS and NS conditions. The chlorophyll index values in the majority of HS-treated cabbage cultivars (except for WCC1) were affected by heat stress and were expectedly reduced as compared to the control plants. In particular, heat stress reduced the chlorophyll content of white cabbage cultivars WCC2 and WCC3 and the red cabbage cultivar RCC as compared to control. Meanwhile, a higher reduction in chlorophyll content was observed in RCC, WCC3 and WCC2 by −1.2-fold, −1.11-fold and −1.11-fold. In addition, an increment in chlorophyll content was observed in WCC1 by +0.9-fold. It is thought that the chlorophyll content in WCC1 was increased under HS conditions because the leaves of WCC1 may respire faster leading to an increase in photosynthesis compared to control. Then, the chlorophyll accumulates in the leaves in higher amounts under stress than in the control. This enables the crop plants to complete their life cycle earlier under stress conditions through physiological adaptation.

The relative reduction of CM1000^TM^ chlorophyll content (%CMCC) caused by HS was evaluated through the comparison of CM1000^TM^ values between NS and HS cultivars. Notably, the highest percentage of reduction in chlorophyll content under HS occurred in the RCC by 26.86%, despite having a considerable value in the CM1000^TM^ chlorophyll index. Additionally, heat stress significantly induced reduction in chlorophyll, and the content of this molecule reduces in WCC2, WCC3 and WCC4 by 16.35%, 11.16% and 7.8%, respectively. Whereas, WCC1 did not show any relative reduction in the content of chlorophyll under HS (Figure 2b).

### 2.3. Effect of Heat Stress on Stomatal Conductance in Brassica oleracea Cultivars

**Figure 3:**
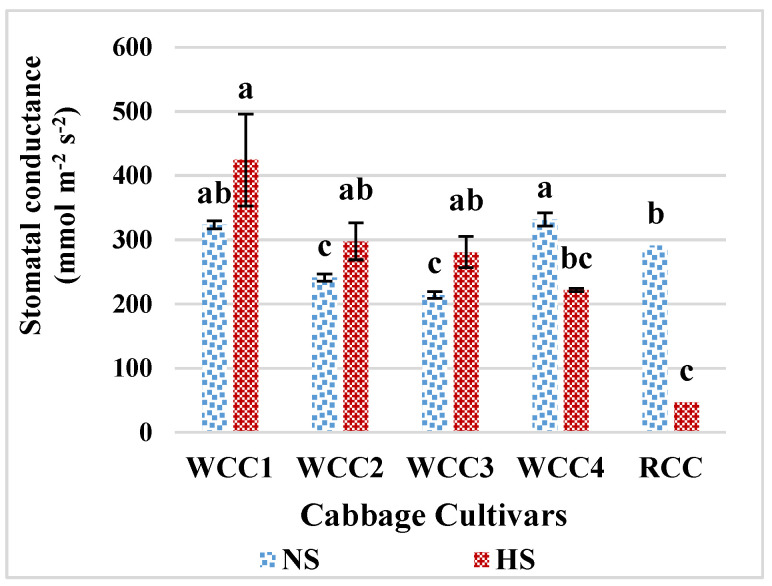
Mean phenotypic expression of stomatal conductance (mmol m^−2^ s^−1^) of five cabbage cultivars under HS (at 42 °C; for 5 h) and NS (25 °C) conditions. Bars denote the mean of at least 4 measurements ± SE. Mean values within each cultivar with different letters are significantly different based on Tukey’s HSD test at *p* < 0.05.

Our results showed that the rate of stomatal conductance for cabbage cultivars in NS ranged from 214 µmol m^−2^ s^−1^ for WCC3 as the lowest rate to 324.4 µmol m^−2^ s^−1^ for WCC1 as the highest rate. Some of the white cabbage cultivars including WCC1, WCC2 and WCC3 when subjected to HS remarkably showed a slight increase in stomatal conductance rates compared to the control condition, apart from WCC4 and RCC, which showed a considerable reduction in stomatal conductance. The increment in stomatal conductance was higher under HS for WCC1 (424.23 µmol m^−2^ s^−1^) (Figure 3). There is a direct correlation between heat tolerance and the ability of plant to sustain leaf gas exchange and CO_2_ assimilation rates under HS [4]. The magnitude of change of stomatal aperture size (opening or closing) in response to high temperatures and boosted CO_2_, along with the effects on associated physiological processes such as *E* and *A*, differed between the cultivars. The interplay between temperature increment resulting in increasing stomatal conductance, and elevation of CO_2_, which leads to decrease stomatal conductance, differed between the cultivars, suggesting that it could contribute to the differences in behaviour among cultivars in the predicted future climate [22]. 

### 2.4. Effect of Heat Stress on Chlorophyll fluorescence in Brassica oleracea Cultivars

**Figure 4:**
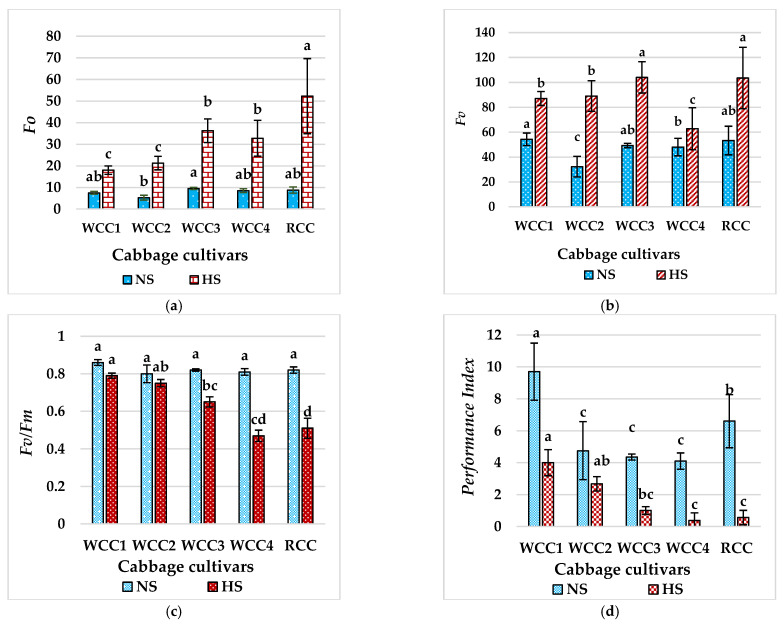
Mean phenotypic expression of chlorophyll fluorescence under HS. (**a**) Comparison of minimum fluorescence (*Fo*) in five cabbage cultivars, (**b**) comparison of variable fluorescence (*Fv*) in five cabbage cultivars, (**c**) comparison of the *Fv/Fm* ratios in five cabbage cultivars measured and (**d**) comparison of photosynthetic performance index (*PI_ABS_*) in five cabbage cultivars. All measurements were performed under HS (at 42 °C; for 5h) and NS (25 °C) conditions. Bars denote the mean of at least 5 measurements ± SE. Mean values within each cultivar with different letters are significantly different based on Tukey’s HSD test at *p* < 0.05.

Since the emitted chlorophyll fluorescence from plant leaves provides a valuable insight into the health of the photosynthetic systems within the leaf, chlorophyll fluorescence has been used as an indicator for HS tolerance. In our experiment, high temperature treatment also modified the chlorophyll fluorescence emission in stressed white and red cabbage cultivars. Thus, in all of these cabbage cultivars, initial fluorescence (*Fo*) values increased (Figure 4a). Meanwhile, the values of maximum chlorophyll fluorescence (*Fm*) were also increased in the treated plants (Figure 4b) at the end of the heat stress in relation to the control treatment. Maximum photochemical efficiency of photosystem II (PSII) in dark-adapted leaves, expressed as *Fv/Fm* (Figure 4c), was reduced in all of the stressed cultivars of white and red cabbages at the end of the heat treatment. Particularly, more significant and higher reduction in *Fv/Fm* was observed in the heat-stressed cabbage cultivars WCC4, RCC and WCC3, with values of 0.47, 0.51 and 0.65 compared to all of the NS cabbage cultivars with their *Fv/Fm* above 0.8. In addition, heat stress noticeably diminished the photosynthetic performance index (*PI_ABS_*) of all the white and red cabbage cultivars, WCC1, WCC2, WCC3, WCC4 and RCC by 58.7%, 43.7%, 77.1%, 90.1% and 91.5%, respectively, compared to control (Figure 4d). The values of the *PI_ABS_* parameter derived from chlorophyll fluorescence records confirmed a much higher responsiveness of *PI_ABS_* compared to *Fv/Fm*. The values in HS treatment were significantly lower in both white and red cabbages in all cultivars. Moreover, the *PI_ABS_* values indicated a lower photosynthetic performance of HS-treated cultivars compared to NS.

### 2.5. Effect of Heat Stress on Cell Membrane Thermostability (CMT) and Relative Thylakoid Damage in Brassica oleracea Cultivars

**Figure 5:**
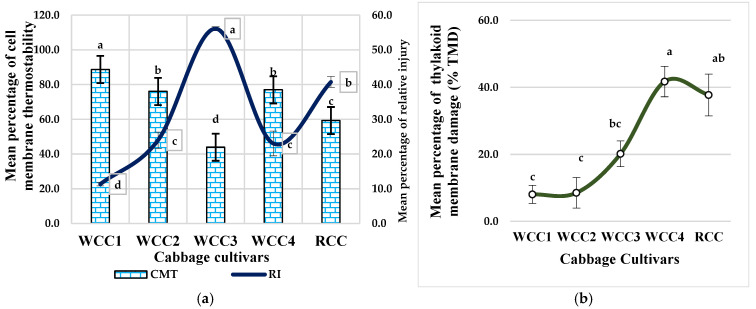
(**a**) Cell membrane thermostability (CMT) index and relative injury as determined by CMT and (**b**) mean percentage of damage to thylakoid membrane of in five cabbage cultivars measured under HS (at 42 °C; for 5 h) and NS (25 °C) conditions. Bars denote the mean of at least 5 measurements ± SE (*n* = 5). Mean values within each cultivar with different letters are significantly different based on Tukey’s HSD test at *p* < 0.05.

CMT values show significant differences between white and red cabbage cultivars (Figure 5a) at the end of the heat treatment, even though CMT values ranged from 43.9% for WCC3 to 88.6% for WCC1. Meanwhile, there were also significant relationships between relative injury (RI) as an index of the CMT of the heat-stressed white cabbage cultivar WCC3 and the red cabbage cultivar RCC by 56.1% and 40.7%, respectively. The CMT test is based on the fact that the injury inflicted on leaf tissues under high temperatures weakens the cell membrane, which leads to a leakage of electrolytes out of the cell [23]. Moreover, consistent with our chlorophyll content measurement results (Figure 2a,b), the reduction in chlorophyll pigment might be the effect of lipid peroxidation of thylakoid membranes and chloroplasts. In order to evaluate the impact of HS on thylakoid membrane damage (TMD), the *F_V_*/*F_M_* values between NS and HS cultivars were compared accordingly. Different cultivars revealed clear differences for relative damage to cell membrane and thylakoid caused at high temperatures (Figure 5b). In particular, the lowest percentage of thylakoid membrane damage due to the high temperatures was recorded for WCC1 at 8% and WCC2 at 8.5% among all heat-stressed cabbage cultivars. This indicates that these cultivars can remain physiologically active for a longer period following experiencing HS. Whereas, heat-shock RCC and WCC4 more significantly demonstrated higher thylakoid membrane damage under high-temperature stress, by 41.7% and 37.7%, respectively.

To summarize, Table 1 is a summary of all the physiological and morphological parameters used to evaluate the effects of high temperatures comprehensively, using indicator values as reference for the selection of heat-tolerant cabbages that have been generated by employing state-of-the-art, precision and useful tools.

### 2.6. Expression Pattern of BoHSP70 and BoSCL13 as Specific Marker Genes for the Heat Tolerance Trait in Cabbage

**Figure 6:**
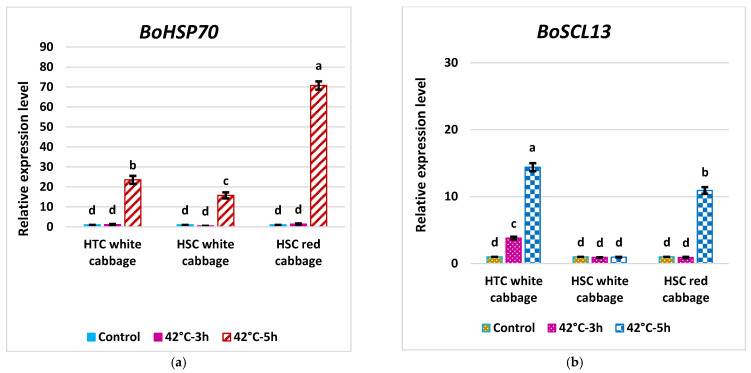
Expression pattern of (**a**) *BoHSP70* and (**b**) *BoSCL13* as specific marker genes for the heat tolerance trait in cabbage cultivars. Four-week-old plants were HS treated at 42 °C for 0, 3 and 5 h. The expression levels at 0h were defined as 1.0 and non-stress condition (control). Bars denote the mean ± SE (*n* = 3). Mean values within each cultivar with different letters are significantly different based on Tukey’s HSD test at *p* < 0.05.

Three biological replicates consisting of one HSC white cabbage (WCC3) and one HTC white cabbage (WCC1) together with one HSC red cabbage (RCC) from the same treatments for the morphophysiological studies mentioned above were used in this study. For the gene expression analysis, the samples were harvested after HS at 42 °C for 3 and 5 h, and for control samples (NS), the seedlings were continually grown at 25 °C. We performed RT-qPCR analyses to evaluate the transcript levels of *BoHSP70* under HS conditions. The expression level of *BoHSP70* in RCC and WCC1 exposed to HS at 42 °C for 3 h showed a slight increase by 1.36-fold and 1.13-fold, respectively, whereas WCC3 showed a slight decrease by 0.45-fold. Continuous incubation of cabbage plants under HS at 42 °C for 5 h resulted in a dramatic increase in the expression of *BoHSP70* in all cultivars. RCC expressed the highest increase in transcript level of *BoHSP70* by 70.7-fold followed by a moderate upregulation in WCC1 (23.5-fold) and WCC3 (15.7-fold) (Figure 6a). The results of this investigation showed that the expression of *BoHSP70* is constitutive and highly enhanced by HS in HSC and HTC, as in *AtHSP70* [2]. The previous Genechip microarray analysis revealed that *BoSCL13* could be a marker to distinguish HTC lines because an increased expression of this gene is thought to be pertinent to heat tolerance and/or good cabbage-head formation at high temperatures [2]. Thus, we carried out RT-qPCR analyses to evaluate the transcript levels of *BoSCL13* under HS conditions to determine the typical heat-induced expression of *BoSCL13* (Figure 6b). Our results revealed that the expression pattern of *BoSCL13* was only induced in WCC1 compared to RCC and WCC3 cultivars at 42 °C for 3 h. Whereas, the *BoSCL13* expression pattern increased in the RCC and WCC1 cultivars but not in WCC3 under HS-treated plants at 42 °C for 5 h. The results of the *BoSCL13* expression pattern found in this investigation are similar to the findings reported by Park et al. (2013) [2].

### 2.7. Analysis of Fold Change in Expression of DPB3-1 and DRIP1 in Heat-Tolerant and Heat-Sensitive Cabbage Cultivars

**Figure 7:**
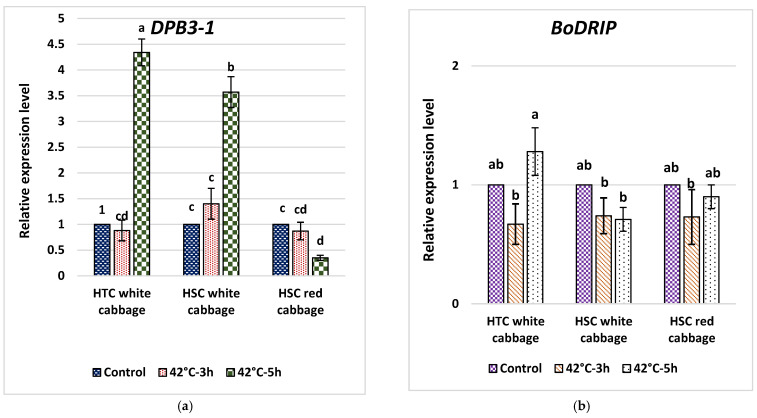
Expression pattern of key heat-stress response transcriptional regulators, (**a**) *BoDPB3-1* and (**b**) *BoDRIP*, in red cabbage compared to heat-tolerant and -sensitive cabbage cultivars. Four-week-old plants were HS treated at 42 °C for 0, 3 and 6 h. The expression levels at 0h were defined as 1.0 and non-stress condition (control). Bars denote the mean ± SE (*n* = 3). Mean values within each cultivar with different letters are significantly different based on Tukey’s HSD test at *p* < 0.05.

We evaluated the transcript levels of *BoDPB3-1* and *BoDRIP1* under HS 42 °C for 3 and 5 h (Figure 7). *BoDPB3-1* showed significantly higher expression at 42 °C for 5 h of HS in WCC1 by 4.3-fold and WCC3 by 3.5-fold compared to NS, whereas the expression pattern of *BoDPB3-1* in HS-treated RCC was relatively downregulated under HS conditions at 42 °C for both 3 h and 5 h by 0.87-fold and 0.35-fold, respectively (Figure 7a). Further, the expression level of *BoDRIP1* was only induced in WCC1 by 1.28-fold under HS conditions at 42 °C for 5 h, whereas the transcript level of *BoDRIP* in WCC3 was decreased during HS at 42 °C for 5 h. The expression pattern of *BoDRIP* was relatively downregulated at 42 °C for 3 h in all cabbage cultivars (Figure 7b).

## 3. Discussion

To mitigate the effects of HS, it is important to screen some indicators (Table 1) to determine cultivars that can withstand environmental challenges. In this study, we evaluated the physiological data, morphological characteristics and the expression levels of key genes in different heat-response pathways influenced by the thermostability of different *B. oleracea* cultivars. In plants, photosynthesis is one of the physiological processes that is greatly affected by heat [24]. The impact of HS is mostly on the photosynthetic capacity of plants, especially C3 plants such as the *Brassica* species compared to C4 plants because of the decline in ribulose-1, 5-bisphosphate carboxylase/oxygenase (rubisco) activity and CO_2_-specificity of rubisco. The reduction in rubisco activity under HS may decrease the capacity of photosystem I to function as an electron receiver from photosystem II (PSII), possibly intensifying the negative effect of HS on PSII [25,26].

### 3.1. High Temperatures Decrease the Content of Chlorophyll in Brassica oleracea Cultivars

Our results clearly demonstrate that leaf chlorophyll content as measured by CM1000™ index values varied between cultivars, but it is uncertain whether cultivars with higher CM1000™ value are heat tolerant. Chlorophyll content is one of the plant physiological characteristics influenced by HS [27]. Our results showed that heat stress reduced the chlorophyll content of most of the white cabbage cultivars and red cabbage cultivars. It is well understood that under HS, the activity of chlorophyll-degrading peroxidase and chlorophyllase rises, and the content of chlorophyll is extremely diminished [28].

### 3.2. High Temperatures Damage the Thylakoid Membrane of the Ultrastructure of Chloroplasts in Brassica oleracea Cultivars

The high sensitivity of photosynthesis to high temperatures and the inconsistency of cellular energy caused by heat damage are irrefutable, which is largely reflected in the discrete modification to the redox state relevant to the injury of thylakoid membranes [28]. The stroma of chloroplasts where carbon metabolism occurs, and the thylakoid lamellae where photochemical reactions take place are considered the primary sites of injury at high temperatures [29,30]. In the present study, candidate cultivars from white and red cabbage species showed clear variations in relative damage to the cell membrane and thylakoid resulting from HS. For example, in our investigation of white cabbage, there are cultivars with the least thylakoid membrane damage due to injury at high temperatures. Most probably, these cultivars can withstand major alterations in chloroplasts under HS, such as an altered structural organization of thylakoids, loss of grana stacking and swelling of grana [31,32]. Heat-tolerant *Brassica* cultivars (WCC1 and WCC2) in this study showed much better cell membrane thermostability compared to heat-sensitive *Brassica* cultivars of the white and red cabbage cultivars (WCC3 and RCC) under HS conditions. The PSII activity was also considerably abated or unexpectedly stopped under high temperatures for both HTC and HSC, consistent with the report by Morales et al. (2003) and Hu et al., (2020) [28,33].

### 3.3. High Temperatures Damage the PSII and REDUCE Fv/Fm in Brassica oleracea Cultivars

Chlorophyll, the key photosynthetic pigment in the chloroplasts’ thylakoid membrane, can intake light energy and stimulate electron transfer during the initial and crucial processes of photosynthesis [34]. However, HS decreases the amount of photosynthetic pigments [30]. *Fv/Fm* is generally used to analyse heat-induced damage to PSII [35], and HS decreases *Fv/Fm* in a variety of plant species [36]. For many plant species, the approximate optimal *Fv/Fm* value ranges from 0.79 to 0.84, with lowered values indicating plant stress [37]. In this study, *Fv/Fm* was reduced in all of the stressed cultivars of white and red cabbages under HS. In particular, less reduction in *Fv/Fm* was observed in HTC of WCC1 and WCC2 (0.79 and 0.75) compared to the other NS cabbage cultivars with their *Fv/Fm* above 0.8.

### 3.4. High Temperature and Its Impact on Stomatal Conductance, Photorespiration and Photosynthesis in Brassica oleracea Cultivars

Stomatal conductance directly alters the relation of plant water and photosynthesis [22]. Heat considerably influences the status of leaf water, intercellular CO_2_ concentration and leaf stomatal conductance. Consistently, in this study the HSCs of *Brassica* species showed a reduction in stomatal conductance as demonstrated by WCC4 and RCC compared to NS plants. Closure of stomata under high temperatures also leads to impaired photosynthesis, affecting the intercellular CO_2_ [31]. Furthermore, with rising temperatures, the rubisco affinity for CO_2_ declines, which boosts oxygenase activities. This results in an increase in photorespiration and reduction in photosynthesis efficiency, eventually reducing crop yields [28]. The decline in chlorophyll pigment is also the result of the lipid peroxidation of chloroplast and thylakoid membranes as shown in cabbage due to HS. Our findings clearly demonstrated that PSII photochemistry (proportion of *F_v_/F_m_*) and leaf stomatal conductance also decrease under HS. The present study shows that HS significantly decreased chlorophyll content (20.5%), *F_v_/F_m_* ratio (27%), *gs* (16%) and CMT (44%) in heat-sensitive *Brassica* cultivars. According to [38], CMT above 60% is an indicator of heat tolerance, 30% to 60% moderate tolerance and less than 30% indicates sensitivity to HS. The mean value obtained for CMT in this study agrees with the work of [23], where CMT was measured in five cabbage cultivars of white and red cabbages. In particular, electrical conductivity has been employed as an indicator of perturbation of membrane permeability to identify heat-tolerant genotypes in *B. oleracea* species [23] and for screening for heat tolerance in different crops [39,40].

### 3.5. GeneMANIA Helps to Predict the Function of Genes and Gene Sets

The application of a bioinformatics tool, GeneMania, enabled a co-expression network to be produced showing how candidate genes of interest in this study interact with each other. GeneMANIA covers 10,244,303 edges between 24,815 *Arabidopsis* genes [41] and also contains a richer network to find associations between genes. GeneMANIA uses a more advanced strategy, taking into account global connectivity between genes and also further exploiting indirect connections in the network [42]. Our GeneMANIA gene–gene interaction network shows that there is about 66.87% of physical interaction among the target genes in this study, including *BoHSP70, BoSCL13, BoDPB3-1* and *BoDRIP1*. *BoDRIP1* acts as an E3 ubiquitin-protein ligase performing as a negative regulator of the response to water stress. It mediates ubiquitination and subsequent proteasomal degradation of the drought-induced transcriptional activator, DREB2A. Our GeneMANIA gene–gene interaction network showed that DREB2A is an intermediator between the *BoHSP70* group and the *BoDRIP1* group, with about 14% of predicted interactions among them. RING1B performs as a putative E3 ubiquitin-protein ligase that mediates mono-ubiquitination of “Lys-119” of histone H2A (H2AK119ub), thereby playing a central role in the histone code and gene regulation. RING1B here is an intermediator in the interaction between the *BoDRIP1* group of genes and the *BoDPB3-1* (NF-YC10) group of genes (Figure S1).

### 3.6. High Temperature and the Phenotypic Differences between HTCs and HSCs

In this study, HTCs showed the most noticeable cabbage head formation compared to HSCs. The HTCs can be differentiated based on their better ability in forming heads at high temperatures [2]. Several reports have shown that cabbage head formation is significantly influence by genotype–environment interaction [11]. In our study, the HTCs began head formation in the vegetative stage, unlike the HSCs. Relevant to the morphological characteristics, the transcript levels of *BoSCL13* were induced in heat tolerant-cabbage lines under HS, suggesting that *BoSCL13* may be involved in heat tolerance and/or better cabbage head formation under HS [2]. Consistent with this report, our results demonstrated that the expression pattern of *BoSCL13* was induced in HTC white cabbage by 3.8-fold compared to HSC red cabbage and HSC white cabbage at 42 °C for 3 h. Our findings support the use of *BoSCL13* as a marker to distinguish HTCs at an early stage following heat treatment. Furthermore, *BoSCL13*, which showed further enhanced expression in HTC white cabbage by 14.4-fold compared to NS but was not expressed in HSC white cabbage at 5 h under HS, can be used to differentiate HTC from HSC in white cabbage cultivars only, as strongly induced expression of this gene was observed in HSC red cabbage (10.91) at 5 h.

### 3.7. Analysis of Fold Change in the Expression of Key Genes in the HS Response Network and Evaluating Potential Molecular Indicators to Differentiate HTC from HSC in Brassica oleracea

Among all the gene–gene interactions, *BoHSP70* appeared to be an important component of the mediator complex. *BoHSP70* acts in cooperation with other chaperones (HSP70s, HSP70-15, HSP70-5, HSP17-7 and DNAJ heat-shock family protein) which are key components that facilitate the folding of de novo synthesized proteins, assist the translocation of precursor proteins into organelles and are responsible for the degradation of damaged proteins under stress conditions with about 12.7% co-expression interaction (Figure S1). Strongly induced expression of *BoHSP70* observed for all three cultivars under HS compared to NS plants at 5h suggested that *BoHSP70* can be used as expression biomarker to monitor HS in both white and red cabbage cultivarsm supporting the findings by Park et al., (2013). The enhanced expression of *BoSCL13* in HSC red cabbage could be due to the interaction of co-expressing SCL13 with HSPs as shown in the gene–gene interaction network (Figure S1) and confirmed by our gene expression analysis of *BoHSP70* and *BoSCL13* in HSC red cabbage. The pigments in the red cabbage belong to a group of compounds called anthocyanins, which are part of a larger group of structures called flavonoids [43]. The role of HSPs in protecting enzymes from denaturation and cellular degeneration is possibly retained with prolonged HS in RCC, most likely due to the high accumulation of pigment and flavonoids [44].

### 3.8. The Expression of BoDRIP as a Negative Regulator Domain of DREB2A Contrary to BoDPB3-1 as a Positive Interactor of DREB2A

Two mechanisms may influence the DREB2A HS response pathway which target the selectivity [18,45] and regulation of protein stability [46,47]. In order to investigate the first mechanism, we studied the expression of *BoDPB3-1,* a DREB2A interactor for enhanced selectivity of HS response genes by DREB2A. *BoDRIP*, which is involved in the 26S proteasome degradation pathway of proteins, was used to study the second mechanism. DPB3-1 (NFYC10) and NF-YB3 are heat stress-inducible genes, and NF-YB3 is transported to the nuclei under HS. Together, DPB3-1, NF-YB3 and NF-YA2 form a trimer which enhances the transcriptional activity of DREB2A. Previous reports elucidated that in the presence of DPB3-1, the stabilized DREB2A induces the expression of HS-responsive genes through binding to a dehydration-responsive element found in their promoters [14]. Our results confirmed that expression of the *BoDPB3-1* gene was induced by HS in both HTC and HSC white cabbages but was supressed in HSC red cabbage. The expression level of *BoDPB3-1* in HSC red cabbage exposed to HS at 42 °C for 3 h and 5 h showed a slight repression by 0.87-fold and 0.35-fold, respectively. The HSC red cabbage plants revealed considerably higher sensitivity to heat than the HTC and HSC white cabbage plants from our physiological measurements and morphological characteristics. The increased sensitivity to heat stress in HSC red cabbage shows an important role of DPB3-1 in the HS response. Since expression of DPB3-1 was not induced in prolonged HS exposure, the trimer involving DPB3-1 cannot form in HSC red cabbage for the activation of DREB2A transcriptional activity. In *Arabidopsis,* the reduced expression of DPB3-1 suppressed the expression of heat stress-inducible genes [15]. To the best of our knowledge, this is the first report of DPB3-1 on gene expression in *B. oleracea* cultivars for screening their HS tolerance.

### 3.9. Opportunities for Crop Improvement for Heat Stress Tolerance

Understanding the expression of HS-responsive genes and the interactions between them may help in the genetic improvement of vegetable crops. HTC will help to meet growing demands for sustainable and safe food production through molecular breeding and genetic engineering. The advent of CRISPR technology and the Cas9-associated protein and its catalytically dead version provide an influential genetic manipulation tool [48] to combat the effects of climate change which can drive forward crop improvement research in response to environmental stresses. This study has contributed to the validation of potential targets based on morpho-physiological measurements, gene expression profiles and the gene network interaction analysis under HS for greater precision in genetic manipulation.

## 4. Materials and Methods

### 4.1. Plant Materials and Growth Conditions

Seeds of two varieties of *Brassica oleracea* including var. *capitate* f. *alba* and var. *capitata* f. *rubra* were selected to conduct this study. Four commercial hybrid cultivars of white cabbage (*Brassica oleracea* var. *capitate* f. *alba*), named KAGAYAKI (WCC1), U. S Hybrid Cabbage (WCC2), PS Petoseed Hybrid Cabbage (WCC3) and Delta Green (WCC4), were used. Additionally, in order to see the variances better in this study, another commercial hybrid cultivar of red cabbage (*Brassica oleracea* var. *capitate* f. *rubra*), named Red Globe (called in this study RCC), was also selected (Table A2). All the hybrid seeds were obtained from the local market at Serdang, Selangor, Malaysia. Germination of soil-grown cabbage seeds was carried out in seedling trays containing sterilized and pasteurized peat moss. Germinated seeds were grown for four weeks, and the soil was kept evenly moist. Cabbage plants were fertilized when the plants were established with a high nitrogen fertilizer such as 10-5-5, N-P-K, in polybags with 6 kg of potting mixture composted with sterilized and pasteurized 2/4-part peat moss, 1/4-part large vermiculite, 1/4-part perlite and irrigated through drip irrigation. The obtained plants were left to grow in a growth chamber at 24 °C for 16 h day and 8 h night cycles with 150 μE m^−2^ s^−1^ light. To examine phenotypic differences between HTCs and HSCs under HS, some cabbage cultivars were left to form cabbage heads at a higher temperature.

### 4.2. Heat Treatments

Four-week-old, young soil-grown cabbages were subjected to HS at 42 °C for 3 h and 5 h (HS) in an incubator or continuously grown in a growth chamber at 25 ± 2 °C (NS) to form cabbage heads.

### 4.3. Chlorophyll Measurements

The chlorophyll content of each plant was measured indirectly by using a hand-held FieldScout CM 1000 ^TM^ Chlorophyll Meter, Aurora, Illinois, USA. The CM1000 chlorophyll meter perceives light at wavelengths of 700 nm and 840 nm to assess the amount of chlorophyll in leaves. At each wavelength, the reflected light is estimated. The chlorophyll index values were measured on different varieties of *B. oleracea,* including white cabbage and red cabbage, when the plants were exposed to HS (at 42 °C, 5 h) compared to NS (control at 25 ± 2 °C) conditions. The fourth opened leaf of each plant of five biological replications of cabbages per cultivar was used to measure the chlorophyll index values. Measurements of chlorophyll were carried out on the adaxial side of cabbage leaf for each plant. Averages of three readings were taken per leaf measurements. The mean value of all assessments was based on five replications obtained through statistical analysis according to Thomason et al. (2018). The relative reduction of CM 1000 chlorophyll content (CMCC) caused by HS was assessed through comparison of the CM1000^TM^ values between plants under control conditions and heat treatments. The relative chlorophyll content reduction was valued as 

% CMCC = [(CM 1000 control − CM 1000 heat)/CM 1000 control] × 100.(1)

### 4.4. Stomatal Conductance Measurements

A leaf porometer (model SC-1, Decagon Devices, Inc., Pullman, WA, USA) was used to measure stomatal conductance (mmol m^−2^ s^−1^) on the abaxial side of the third fully expanded leaves of five biological replications of cabbages per cultivar at ± 10% accuracy (*n* = 4). Measurements of stomatal conductance were taken early in the morning (8–10 a.m.) for the control cabbage plants and 24 h after heat stress for the heat-treated plants. Instrument calibration was performed before each set of measurements based on the manufacturers’ guidelines.

### 4.5. Chlorophyll Fluorescence (CF) Test

CF measurements were performed using a Handy-PEA chlorophyll fluorimeter device (Plant Efficiency Analyser, Hansatech instruments Ltd., Norfolk, UK). The photosynthetic efficiency was determined based on the value obtained for *Fv/Fm*, whence *Fv* = *Fm* − *Fo*, as variable fluorescence in the dark-adapted state. Here, *Fo* is the minimal fluorescence state and is explained as the point where all antenna sites are not closed. The antenna complex is a light-harvesting membrane-associated aggregate of proteins and photosensitive pigments such as chlorophyll and carotenoids. They are situated inside the chloroplasts of photosynthetic organisms, capture the energy from light and transfer it to the reaction centre where chemical reactions take place [49]. *Fm* (Maximal fluorescence) arises when all sites of the antenna are closed under a light saturation flash. Damage to the light harvesting complexes is expressed as a decrease in *Fv/Fm.* To determine *Fo*, dark adaptation was performed using dark adaptation clips. These clips were attached to the leaves of five biological replications of cabbages per cultivar for half an hour prior to taking the measurements at midday and allowing emission measurements at a uniform distance while a sliding shutter excluded the light. The light pulse intensity used was 3500 µmol m^−2^ s^−1^ for 1 s [50]. CF transient data were used to perform all of the calculations. The maximum quantum yield (efficiency) of PS II photochemistry (*Fv/Fm*) and the performance index (*PI_ABS_*) were then further evaluated and analysed to differentiate the most heat-tolerant and -sensitive cabbage cultivars. CF, the proportion of variable (*F_V_* to maximum fluorescence *F_M_*), was utilized as an indirect method to measure the damage to the thylakoid membrane [51,52]. To measure thylakoid membrane damage (TMD) caused by HS, the *F_V_/F_M_* values between the control and heat-treated plants were compared. The relative damage was estimated as 

%TMD = [(Fv/Fm control − Fv/Fm heat)/Fv/Fm control] × 100.
(2)

### 4.6. Cell Membrane Thermostability (CMT) Test

CMT measurement was conducted according to the procedure demonstrated by Usman et al. (2015). Samples with similar leaf sizes were carefully chosen from different white and red cabbage cultivars under NS (control at 25 ± 2 °C) conditions. A 5 mm diameter leaf puncher was used to prepare six leaf disks, each approximately 5mm in size, consisting of a paired set (control (C) and treatment (T)). The disks were punched from five biological replications of fully expanded third leaves from each white and red cabbage cultivar and each was replicated three times. Prior to performing the test, to eliminate electrolytes adhering to the leaf discs surface, the paired set of leaf disks were set in two distinct test tubes (50 mL) and immersed thoroughly with four changes of deionized water (DIW), 10 mL each time. Then, both sets of test tubes were embedded into 10 mL of DIW and sealed with aluminium foil to avoid evaporation. Next, test tubes containing the C set were kept at toom temperature. The test tubes containing the treatment set were incubated for 20 min at 50 °C in a water bath. Afterwards, both test tubes were kept in the refrigerator. After incubation at 4 °C for 24 h, the test tubes were allowed to reach to room temperature (RT) and initial conductance readings were carried out for both the TEC 1 and CEC 1 sets using a bench electrical conductivity meter (Starter 3000C; Ohaus Corp., Parsippany, NJ, USA). The tubes were then wrapped once more with aluminium foil and autoclaved for 20 min at 121 °C and 0.15 MPa to completely kill the leaf tissue. After autoclaving was accomplished, the tubes wee allowed to cool down to RT and the contents were thoroughly mixed. The reading of the final conductance of TEC 2 and CEC 2 was recorded. The CMT was calculated using following equations:

CMT (%) = (1− (TEC 1/TEC 2))/(1 − (CEC 1/CEC 2)) × 100(3)

where TEC and CEC are the conductance measurements in the treated and control test tubes, respectively, for the initial (CEC 1 and TEC 1) and final (CEC 2 and TEC 2) conductance measurements. Relative injury was also calculated based on the following equation:

%RI= {1 − [1 − (TEC 1/TEC 2)]/[1− (CEC 1/CEC 2)] × 100}.(4)

### 4.7. Determination of the Morphological Characteristics of Heat-Stressed Brassica oleracea

The sampling for morphological analysis of all HS-exposed white cabbage cultivars and one red cabbage cultivar was carried out immediately after giving the HS treatment. The effects of HS on growth performance of the cabbage cultivars were assessed based on the ability to form heads as well as the weight, width and length of the heads that formed and the ratios of the obtained measurements. Measurements of the morphological characteristics were taken in five replications (biological replicates).

### 4.8. Quantitative Real-Time PCR (qRT-PCR) Analysis 

Total RNA was isolated from leaf tissue using TRIzol (Life Technologies, Invitrogen™, CA, USA catalog number: 15596018) according to Macrae’s protocol [53]. iScript cDNA Synthesis Kit (Bio-Rad Laboratories Inc., Hercules, CA, USA) was used in the in vitro transcription of RNA according to the manufacturers’ instructions. RT-qPCR was carried out utilising a Bio-Rad CFX96 real-time device (C1000 Touch thermal cycler) with two primers for each target gene (the list of all primers used in this study is shown in Table A1, Appendix A). For amplification, iTaq Universal SYBR Green supermix (Bio-Rad Laboratories, Inc., Hercules, CA, catalog number: 172-5121) was employed in a final volume of 10 μL. The cycler was programmed as follows: 95 °C for 10 min followed by 40 cycles of 95 °C for 15 s, 60 °C for 1 min, and then 95 °C for 15 s. The reference genes used were *B. oleracea* ubiquitin conjugating enzyme (*BoUBC*), hypothetical protein (*BoUNK1*) and actin (*BoActin*) to normalize the gene expression values. The ΔΔCq method was used to determine the difference in relative gene expression of the candidate genes in this study. Assessment in RT-qPCR was performed with three repetitions (technical replicates) of three individual samples (biological replicates) with all the genes in all the treatments.

### 4.9. Statistical Analysis

Statistical analysis was performed using the statistical analysis system (SAS) program (Version 9.2). All values were shown as mean ± SE (Standard Error); (*n* = 5) represents means for five biological replications per treatment. Data were subjected to one-way analysis of variance (ANOVA) for mean comparison and significant differences were calculated according to Tukey’s Studentized Range (HSD) Test. Probability level for all statistical analyses was 0.05.

### 4.10. Drawing Co-Expressed Gene Networks for Query Genes in This Study through GeneMANIA Tools

The key genes in the HS response network that were used for expression profiling in this study were nominated for the network study in abiotic stress tolerance. In order to depict DPB3-1 regulatory networks and the analysed co-expressed genes, the GeneMANIA (http://www.genemania.org accessed August 2019) web tool was used. GeneMANIA is linked to the Arabidopsis model organism database (TAIR) and to the Arabidopsis resource BAR and uses a combination of different datasets to find the genes that are most related to a set of query genes. The combined dataset consists of genetic interactions, pathways, co-expression, co-localization and protein domain similarity. Using this database, an analysis of key genes in HS response interacting with *DPB3-1* together with gene function predictions was carried out.

## 5. Conclusions

In conclusion, the results obtained from our physiological data on chlorophyll content and fluorescence, stomatal conductance, cell membrane thermostability and relative thylakoid damage together with morphological data on cabbage head formation provided a comprehensive universal morpho-physiological indicator for differentiation of heat-tolerant and -sensitive populations of *B. olearacea* cultivars and possibly for other vegetable crops. Our results confirmed that the expression of *BoHSP70* can be a universal biomarker for monitoring HS in cabbages, while *BoSCL13* expression is helpful to differentiate HTC from HSC for white cabbages. The suppression of *BoDPB3-1* expression may reduce selectivity for HS response target genes by DREB2A, resulting in increased sensitivity to HS in HSC red cabbages. Clearly, prior knowledge of cabbage-specific gene expression profiles under HS is essential in applying the gene expression biomarkers based on HS response network.

## Figures and Tables

**Table 1:** A summary of physiological and morphological parameters evaluated in this study can be used as indicators for the selection of heat tolerance in cabbage.

Paramaters	Heat Tolerance Indicators		
	Heat Tolerance	Moderate Tolerance	Sensitive
Reduction of chlorophyll content	X < 10	X = 10.0–20.0	X > 20.0
Stomatal conductance	X > 300	X = 200–300	X < 300
*Fv*/*Fm*	X > 0.75	X = 0.6–0.75	X < 0.6
Cell membrane thermostability	X > 60%	X = 30–60%	X < 30%
Head formation	Yes	Yes	No

## Data Availability

The data are contained within the article.

## Supplementary Materials

The following are available online at https://www.mdpi.com/article/10.3390/plants10061064/s1, Figure S1: Gene interaction network in *Arabidopsis* for the genes of interest used for the gene expression study in this work. The network was generated by the GeneMANIA prediction server [43]. The right panel presents the different types of interactions with respective color coding, depicted with lines connecting genes in the network: physical interactions (pink), predicted (orange), co-expression (purple) and shared protein domains. Red-filled circles considered heat-shock protein binding.

## Appendix A

**Table A1:** List of primers used in this study.

Name	Primer 5’--3’	Reference
UBCF	TTATGAAGGCGGAGTGTTT	[54]
UBCR	TGAACCCTCTCGCATCTC	
UNK1F	TTACACACCACAAAGAGAGT	[54]
UNK1R	TACAACACCTGAAACCCATG	
ACT F	GAATCCACGAGACAACATAT	[54]
ACT R	AGGGAAGCAAGAATGGAAC	
Hsp70 F	GGGAAGGCTGTCCAAGGAAGAG	[2]
Hsp70 R	CCATGCCACCAGCTCCACCCATATC	
SCL13 F	CCGAAACTCGTGACGCTAGTGGAGC	[2]
SCL13 R	GCGGAACATGTAGCCATGGGTCTCC	
DPB3 F	CGACGAGAGATACGAGTTCC	Designing specific primers
DPB3 R	GCTTCTTTCCCATTCCTCCA	
DRIP F	TGAAACACGAGAGGAGAAAGG	Designing specific primers
DRIP R	TGGAATCTGAGGCAAGGATG	

**Table A2:** List of the *B. oleracea* cultivars used in this study.

Classification	Source of Seeds	Characteristics
White Cabbage	KAGAYAKI	Mikado Kyowa seed Round shaped head with bright green color. Extra early maturity of 43–48 days, 1.0–1.3 kg in weight. Compact plant with semi-open, compact outer leaves suitable for density planting. Tolerant to Cabbage Yellows (B type) and Black Rot.
	P S Hybrid Cabbage Petoseed	Early maturity tropical type with excellent yield, uniformity, disease resistance, shippability. Resistance: yellows, Xanthomonas campestris.
	Green World Cabbage	High weather conditions. One round shape and average weight of 1.5 kg
	Hybrid Cabbage giant Takii & co., LTD Kyoto, Japan	Hybrid, midseason, Bravo class, denser heads, bright green. Resistance: yellows; high tolerance to black rot, tolerance to thrips, tipburn, heat, cold.
Red Cabbage	Red Globe Mikado Kyowa Seed	Hybrid, round shaped head with deep purplish red color. Medium maturity of 75 days, 1.5–2.0 kg in weight. Uniform, compact, short cored head with thick and tender leaves. Good quality and taste. Slow bursting.
