# Supplementary material for: The Impact of Heat Stress on Morpho-Physiological Response and Expression of Specific Genes in the Heat Stress-Responsive Transcriptional Regulatory Network in Brassica oleracea
Source: Plants (Basel). 2021 May 26;10(6):1064. doi: 10.3390/plants10061064 (PMC8230129; doi:10.3390/plants10061064)
Supplement: Supplementary file 1 [file plants-10-01064-s001.zip › plants-1116073-SI.pdf]

plants

MDPI

Article

The Impact of Heat Stress on Morpho-Physiological Response and Expression of Specific Genes in the Heat Stress-Responsive Transcriptional Regulatory Network in *Brassica oleracea*

Mahdi Moradpour [1], Siti Nor Akmar Abdullah [1,2,*] and Parameswari Namasivayam [3]

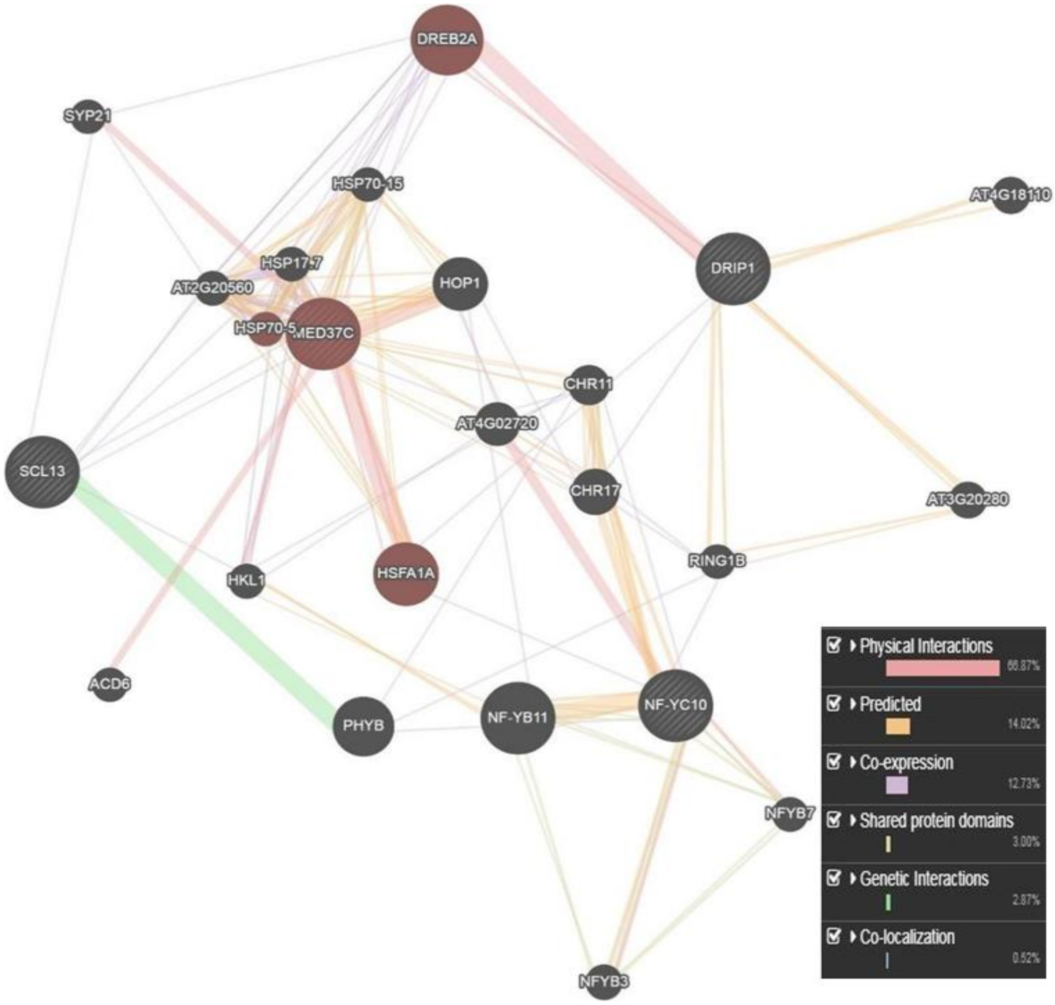

Figure S1. Gene interaction network in *Arabidopsis* for the genes of interest used for the gene expression study in this work. The network was generated by the GeneMANIA prediction server [44]. The right panel presents the different types of interactions with respective color coding, depicted with lines connecting genes in the network: physical interactions (pink), predicted (orange), co-expression (purple) and shared protein domains. Red-filled circles considered heat-shock protein binding.
